# Enhancing Monacolin K and GABA Biosynthesis in *Monascus pilosus* via *GAD* Overexpression: Multi-Omics Elucidation of Regulatory Mechanisms

**DOI:** 10.3390/jof11070506

**Published:** 2025-07-04

**Authors:** Wenlan Mo, Yiyang Cai, Simei Huang, Lishi Xiao, Yanfang Ye, Bin Yang, Chan Zhang, Zhiwei Huang

**Affiliations:** 1 Engineering Research Centre of Fujian-Taiwan Special Marine Food Processing and Nutrition (Ministry of Education), Fujian Agriculture and Forestry University, Fuzhou 350002, China; mowenlan2024@163.com (W.M.); cai10404@126.com (Y.C.); huangsm0112@163.com (S.H.); xiao_lishi5258@163.com (L.X.); yyf3170@163.com (Y.Y.); yang_bin2024@163.com (B.Y.); 2 College of Food Science, Fujian Agriculture and Forestry University, Fuzhou 350002, China; 3 Fujian Provincial Key Laboratory of Quality Science and Processing Technology in Special Starch, Fujian Agriculture and Forestry University, Fuzhou 350002, China; 4 School of Food and Health, Beijing Technology & Business University (BTBU), Beijing 100048, China

**Keywords:** *Monascus pilosus*, *Glutamate decarboxylase* gene, monacolin K, γ-aminobutyric acid, overexpression

## Abstract

*Monascus* produces various bioactive compounds, including monacolin K (MK), γ-aminobutyric acid (GABA), and *Monascus* pigments (MPs). Studies have shown that overexpressing genes within the MK biosynthetic cluster significantly enhances MK production. Additionally, MK synthesis in *Monascus* is regulated by other genes. Based on previous transcriptomic analyses conducted in our laboratory, a significant positive correlation was identified between the expression level of the *GAD* gene and MK production in *M. pilosus*. In this study, the *GAD* gene from *M. pilosus* was selected for overexpression, and a series of engineered *M. pilosus* strains were constructed. Among the 20 PCR-positive transformants obtained, 13 strains exhibited MK production increases of 12.84–52.50% compared to the parental strain, while 17 strains showed GABA production increases of 17.47–134.14%. To elucidate the molecular mechanisms underlying the enhanced production of MK and GABA, multi-omics analyses were performed. The results indicated that *GAD* overexpression likely promotes MK and GABA synthesis in *M. pilosus* by regulating key genes (e.g., *HPD*, *HGD*, and *FAH*) and metabolites (e.g., α-D-ribose-1-phosphate, β-alanine) involved in pathways such as tyrosine metabolism, phenylalanine metabolism, the pentose phosphate pathway, propanoate metabolism, and β-alanine metabolism. These findings provide theoretical insights into the regulatory mechanisms of MK and GABA biosynthesis in *Monascus* and suggest potential strategies for enhancing their production.

## 1. Introduction

*Monascus* species is a traditional filamentous fungus renowned for its dual-purpose use in food and medicine. It is widely employed in the solid-state fermentation of various grains, such as rice, buckwheat, coix seed, and barley, to produce diverse bioactive secondary metabolites. These include monacolin K (MK), γ-aminobutyric acid (GABA), and *Monascus* pigments (MPs) [1,2,3]. Among these, MK is a key component in *Monascus* fermentation products, known for its ability to reduce cholesterol levels in humans [4,5,6]. Additionally, MK provides protective effects against Parkinson’s disease and neurofibromatosis type I [7] and induces apoptosis in glioblastoma U251 cells [8] among other benefits. Due to its physiological significance, increasing MK production has become a critical goal in the industrial fermentation of *Monascus*. Therefore, researchers have been committed to enhancing the production of MK in *Monascus* species. For instance, Zhang et al. [9] improved MK yield by enhancing *Monascus* mycelium permeability using L-glutamic acid, while Ye et al. [10] achieved higher MK yields by optimizing liquid fermentation media and applying X-ray irradiation.

The biosynthesis of monacolin K (MK) is predominantly governed by the polyketide synthase (PKS) pathway in *Monascus* species, a complex biochemical process that requires the coordinated expression of multiple genes within the associated gene cluster, along with the utilization of key metabolic precursors, including acetyl-CoA and malonyl-CoA [11]. Mechanistically, PKS catalyzes the condensation of acetyl-CoA and malonyl-CoA to assemble the polyketide carbon skeleton of monacolin K. This intermediate then undergoes a cascade of enzymatic transformations, such as oxidation and dehydration, to produce monacolin L. The final step in the biosynthetic pathway involves the action of monooxygenase, which facilitates the conversion to MK [12]. However, challenges such as long fermentation cycles and limited MK yields constrain its applications. Recent advances in fungal secondary metabolism have highlighted genetic modification as a promising approach to overcome these limitations [12,13]. Overexpression of key genes in the MK biosynthetic cluster, such as *mokE* [14], *mokH* [15], and *mokI* [16], has been shown to significantly enhance MK production. Moreover, MK synthesis in *Monascus* is regulated by additional genes. For example, Zhang et al. [17] reported a 48.6% increase in MK production by overexpressing the global regulator gene *LaeA* in *M. purpureus*. Shi et al. [18] enhanced MK and MP production in *M. purpureus* M1 through the overexpression of the *lip10* gene associated with fatty acid synthases. Li et al. [13] improved MK production in *M. pilosus* MS-1 by overexpressing the acetyl-CoA carboxylase gene and the histone deacetylase gene, achieving relative increases of 43.9% and 36.1%, respectively, while shortening the production cycle from 20 days to 14 days. These studies collectively indicate that targeted regulation of key genes can effectively improve MK production in *Monascus* species. However, current research predominantly focuses on genes directly associated with the MK biosynthetic pathway (e.g., the polyketide synthase [PKS] gene cluster), while insufficient attention has been paid to other potential regulatory genes within the broader metabolic network.

Transcriptomic analyses previously conducted in our laboratory revealed a significant positive correlation between MK accumulation and the expression of genes such as glutamate decarboxylase (GAD) and sugar transporters during *Monascus* fermentation. GAD, a pyridoxal-5’-phosphate-dependent enzyme, catalyzes the decarboxylation of L-glutamate to GABA with the release of CO_2_. This enzyme is widely distributed in both eukaryotes and prokaryotes and serves as a key rate-limiting enzyme for GABA synthesis, which plays an important role in amino acid metabolism [19]. In microbial systems, GAD-mediated conversion of glutamate to GABA not only regulates intracellular pH homeostasis but also modulates nitrogen flux and energy metabolism. Furthermore, GABA biosynthesis interfaces critically with secondary metabolite production. Glutamate serves dual functions: as the direct precursor for GABA synthesis and as a key nitrogen donor for multiple secondary metabolic pathways. Consequently, regulation of GAD activity influences not only GABA yield but also indirectly orchestrates secondary metabolite biosynthesis through nitrogen resource allocation [20,21]. GABA itself acts as a neurotransmitter and exhibits physiological functions such as improving memory, alleviating pain, regulating lipid levels, reducing blood pressure, and managing diabetes [22,23].

This study aims to construct a *Monascus* engineering strain overexpressing the *GAD* gene to enhance MK and GABA production. Furthermore, multi-omics analyses will be employed to elucidate the molecular mechanisms by which *GAD* overexpression promotes high-yield MK and GABA production in *Monascus*.

## 2. Materials and Methods

### 2.1. Strains and Media

*M. pilosus* CICC 5045 was obtained from the China Center of Industrial Culture Collection. This strain was originally isolated from fermented cereals (sorghum and barley). Competent *Escherichia coli* strain Top10 and *Agrobacterium tumefaciens* strain AGL-1 were preserved in our laboratory.

LB and YEB media were prepared as described in Reference [24] and used for constructing and transforming the *GAD* gene eukaryotic expression vector into *A. tumefaciens*.

Malt juice medium (Qingdao Hi-Tech Industrial Park Hope Bio-Technology Co., Ltd., Qingdao, China) was used for activating and liquid fermentation culturing of *M. pilosus*.

AIM medium, AIM induction medium, and selection medium were prepared as described in Reference [16] and used for *M. pilosus* transformation via the *Agrobacterium*-mediated method.

Seed liquid medium was prepared as described in Reference [9] and used for inoculum preparation.

Solid-State Fermentation Medium: 30 g of buckwheat was placed into a 350 mL plastic fermentation bottle, and 20 mL of a glycerol-water mixture (1:3, *v*/*v*) was added for solid-state fermentation of *M. pilosus*.

### 2.2. Construction of GAD Eukaryotic Expression Vector

The full-length cDNA sequence of the *GAD* gene from *M. ruber* was obtained from the USDA JGI database (*M. ruber* NRRL 1597 v1.0, https://mycocosm.jgi.doe.gov/Monru1/Monru1.home.html, accessed on 1 August 2023) and synthesized by Biosune Biotechnology (Shanghai, China). The synthesized gene was cloned into the pNeo-0380 vector using the method described in Reference [16] to construct the recombinant plasmid pNeo-GAD (Figure S1). The pNeo-0380 vector was generously provided by Associate Professor Long from Jiangxi Science and Technology Normal University, a previous research collaborator of the author of this article. The construction method of this vector was detailed in the literature [25]. This plasmid pNeo-GAD was transformed into *E. coli* Top10 competent cells. Transformants were verified by restriction enzyme digestion with Hind III and Sac I, following the manufacturer’s protocol.

Primers for verification were designed using Primer3web version 4.1.0 (https://primer3.ut.ee/, accessed on 20 November 2023) based on the nucleotide sequences of the *GAD* gene and the *PgpdA* promoter: GAD-W1C (5′-CGCGTCCGAATATCATCATGAA-3′) and PgpdA-F3 (5′-ACTTCATCGCAGCTTGACTAAC-3′). Primers were synthesized by Fuzhou Biosune Biotechnology Co., Ltd. (Fuzhou, China). PCR verification of *E. coli* transformants was performed as described in Reference [16], using the transformant culture as a template.

### 2.3. Transformation of M. pilosus via Agrobacterium-Mediated Method

The recombinant expression vector pNeo-GAD was introduced into *A. tumefaciens* AGL-1 Via the freeze–thaw method [24]. PCR and restriction digestion were performed as described in Section 2.2 to confirm the recombinant *Agrobacterium* strain pNeo-GAD/AGL-1. *M. pilosus* was then genetically transformed using the *Agrobacterium*-mediated method. After co-culturing *M. pilosus* with *A.* *tumefaciens* and conducting screening cultures, transformants were selected under the following conditions: a layer of YEB screening medium containing 80 μg/mL Geneticin, 200 μmol/L Cefotaxime, and 0.2% Triton X-100 was overlaid on AIM medium. The cultures were incubated at 30 °C for 5–8 days to identify resistant transformants. Following three successive rounds of screening and subculturing, integration of the *GAD* gene was verified by PCR using primers PgpdA-F3/GAD-W1C.

### 2.4. PCR Verification of M. pilosus Transformants

Resistant transformants obtained from Section 2.3 were inoculated in malt juice liquid medium and cultured at 28 °C, 220 rpm for 2–3 days. The original strain CICC 5045 served as a negative control. Genomic DNA was extracted from the mycelia of transformants using the Genomic DNA Rapid Extraction Kit (Beijing Dingguo Changsheng Biotechnology Co., Ltd., Beijing, China). Using genomic DNA as a template, PCR verification was conducted with primers GAD-W1C and PgpdA-F3, as described in Reference [16].

### 2.5. Solid-State Fermentation of Transformant Strains

Positive transformant strains (TG01–TG20) and the original strain CICC 5045 were inoculated on malt juice medium plates and cultured at 28 °C for seven days. The cultures were then transferred to seed medium and shaken at 180 rpm, 28 °C for 48 h. The conidia were collected using sterile lens paper and diluted in sterile water to a concentration of 1 × 10^6^ spores/mL.

Spore suspensions were inoculated into solid-state fermentation medium (10% inoculation volume), with four replicates for each strain. Fermentation was carried out at 28 °C for 12 days. The fermented buckwheat (*Monascus*-fermented buckwheat, MFB) samples were dried to constant weight at 55 °C and ground into powder for further analysis.

### 2.6. HPLC Detection of MK Content in Transformant Strains

Sample Preparation: following the protocol outlined in Reference [16]. Weigh 1.0 g of MFB powder into individual 10.0 mL centrifuge tubes. Adjust the volume to the 10.0 mL mark with 75% (*v*/*v*) ethanol solution. Vortex-mix thoroughly and subject to ultrasonic extraction at 30 °C for 30 min, with intermediate vortex-mixing at 15 min intervals. Following extraction, vortex-mix again and allow the mixture to stand. Subsequently, filter the supernatant through a 0.22 μm microporous membrane prior to HPLC analysis for MK quantification. Both the acid and lactone forms of MK were quantified, and the MK content (mg/kg) was calculated using the formula: standard sample concentration × (sample chromatographic peak area/standard sample peak area) × fixed volume/mass of the tested sample.

### 2.7. HPLC Detection of GABA Content in Transformant Strains

Sample Preparation: Following Xiong et al. [26], 1.0 g of MFB powder was sonicated in 10 mL of 80% ethanol for 30 min, with two extractions. The combined supernatant was adjusted to 25 mL.

Preparation of GABA Stock Solution: Precisely weigh 10 mg of the GABA standard, dissolve in acetonitrile, and dilute to 10 mL to obtain a 1000 mg/L standard solution. Accurately pipette the standard solution to prepare five standard working solutions with concentrations of 1 mg/L, 5 mg/L, 25 mg/L, 60 mg/L, and 100 mg/L. Prepare fresh solutions as necessary.

GABA Derivatization: For derivatization, mix 1 mL of sample solution with 0.2 mL sodium bicarbonate and 0.4 mL 4-dimethylaminoazobenzene-4-sulfonyl chloride in a sealed tube. Incubate at 70 °C for 20 min, then cool to room temperature. Filter the solution through a 0.22 µm organic filter to prepare HPLC detection samples for TG01-TG20 and the *M. pilosus* control strain CICC 5045.

HPLC Conditions: Detection was performed on a Waters e2695 HPLC system using a SunFire C18 column. The mobile phase was acetonitrile and trisodium acetate solution (35:65, *v*/*v*) at a flow rate of 1.0 mL/min. UV detection was set at 438 nm.

GABA content (mg/kg) was calculated using the formula: GABA determination concentration in the sample (mg/L) × sample volume (mL)/mass of the sample (g).

### 2.8. Multi-Omics Analysis of High MK and GABA Yield in Transformant Strains

#### 2.8.1. Transcriptomic Analysis

Based on high-performance liquid chromatography (HPLC) analysis, the strain TG08 was selected from 20 transformed strains for demonstrating significant improvements in both MK and GABA production, exhibiting a 47.5% relative increase in MK yield and 91.1% relative increase in GABA yield compared to the parental strain. Consequently, TG08 was chosen as the overexpression strain for transcriptomic and metabolomic analysis.

Following the solid-state fermentation method described in Section 2.5, the transformant strain TG08 with high MK and GABA yields was selected, with CICC 5045 serving as the control. Fermentation was carried out for 4, 8, and 12 days, with three replicates per treatment. Since the samples used in this study to detect MK and GABA yields were solid-state fermentation products, samples from solid-state fermentation instead of submerged fermentation were employed for transcriptomic analysis to more accurately reflect the regulatory mechanisms of MK and GABA biosynthesis. However, the mycelium from solid-state fermentation could not be completely separated from the fermentation products. Therefore, after fermentation, myceliums were separated from the fermentation product following Wang et al. [27]. Total RNA was extracted from the myceliums of TG08 and the control strain CICC 5045, as described by Huang et al. [5], and transcriptome analysis was conducted. Differentially expressed genes were identified using thresholds of FDR < 0.05 and FC ≥ 1.2 or FC ≤ 1/1.2.

#### 2.8.2. Metabolomic Analysis

Samples of 50 mg each from TG08 and the original strain CICC 5045 were prepared from MFB. Three replicates were used for each treatment. Samples were preprocessed following Huang et al. [16], analyzed via LC-MS, and subsequently subjected to metabolomics analysis of the resulting data. Differential metabolites were identified with thresholds of FDR < 0.05, VIP > 1, and |log2FC| ≥ 1.

#### 2.8.3. Integrated Omics Analysis

Transcriptomic and metabolomic data were integrated using the Majorbio’s cloud platform (www.majorbio.com). Key metabolic pathways affected by *GAD* overexpression were identified, and mechanisms behind enhanced MK and GABA production were elucidated as described by Han et al. [28].

### 2.9. Data Processing and Statistical Analysis

All experiments in this study were conducted with ≥3 biological replicates. Experimental data are presented as mean ± standard deviation (Mean ± SD). Statistical analysis was performed using IBM SPSS Statistics 27.0 software, with a significance threshold of *p* < 0.05 indicating significant differences between treatments. Bar charts depicting MK and GABA content were generated using Origin 24 software, with different letters indicating statistically significant differences among treatments.

## 3. Results and Analysis

### 3.1. Acquisition of GAD Gene Overexpression Strains

#### 3.1.1. Construction of the Eukaryotic Expression Vector for the *GAD* Gene

As shown in Figure S2, PCR amplification and enzyme digestion confirmed the expected band sizes for the recombinant plasmid pNeo-GAD, demonstrating the successful construction of the eukaryotic expression vector pNeo-GAD for the *GAD* gene.

#### 3.1.2. Transformation of *Monascus pilosus* via Agrobacterium-Mediated Method and PCR Verification

The recombinant vector pNeo-GAD was successfully introduced into *Agrobacterium* strain AGL-1, as confirmed by enzyme digestion and PCR verification (Figure S2). Using *Agrobacterium*-mediated transformation, 88 resistant transformants of *Monascus pilosus* were obtained. PCR results (Figure S3) identified 20 positive transformants (TG01–TG20).

### 3.2. MK Content in Transformed Strains (HPLC Detection)

**Figure 1:**
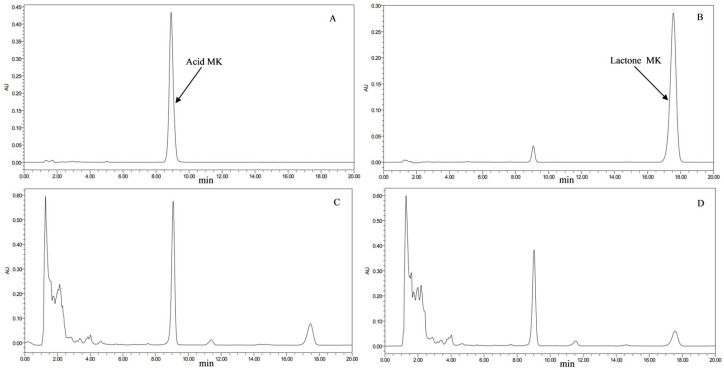
HPLC chromatograms of MK from transformed and original strains of *M. pilosus*. (**A**) Acidic form of MK standard; (**B**) lactone form of MK standard; (**C**) sample from transformed strain of *M. pilosus*; (**D**) sample from original strain of *M. pilosus*.

**Figure 2:**
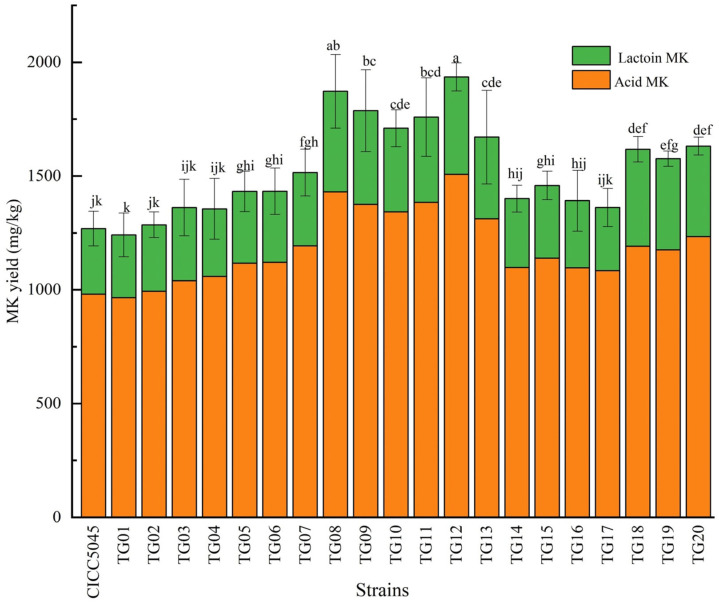
MK yield of transformed and original strains of *M. pilosus*. Different letters among strains indicate significant differences (*p* < 0.05).

HPLC chromatograms (Figure 1) illustrated the MK profiles of the transformed strains and the original strain. As shown in Figure 2, 13 of the transformed strains (TG05, TG06, TG07, TG08, TG09, TG10, TG11, TG12, TG13, TG15, TG18, TG19, TG20) exhibited significantly higher MK production compared to the original strain, with the highest increase observed in TG12 (52.5%), followed by TG08 (47.51%). The other seven transformants showed no significant differences in MK production compared to the original strain.

### 3.3. GABA Content in Transformed Strains (HPLC Detection)

**Figure 3:**
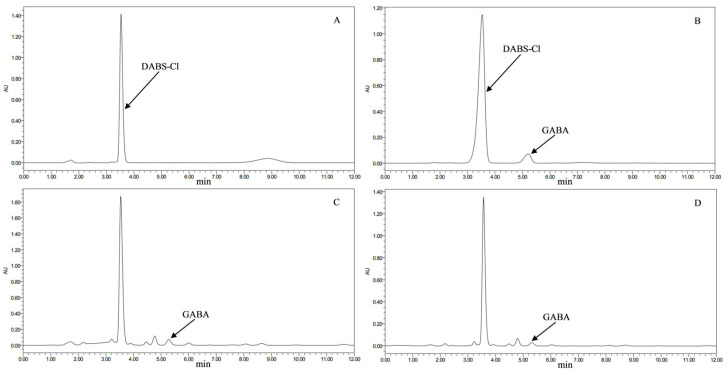
HPLC chromatograms of GABA from transformed and original strains of *M. pilosus*. (**A**) Derivatization reagent DABS-Cl solution; (**B**) GABA standard; (**C**) sample from transformed strain of *M. pilosus*; (**D**) sample from original strain of *M. pilosus*.

**Figure 4:**
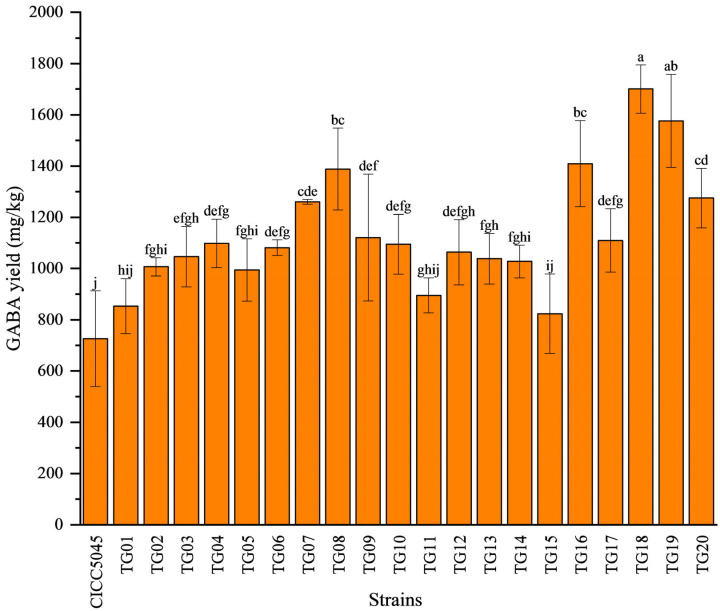
GABA yield of transformed and original strains of *M. pilosus*. Different letters among strains indicate significant differences (*p* < 0.05).

Figure 3 confirmed retention times of the derivatization reagent DABS-Cl (Rt = 3.572 min) and the GABA standard (Rt = 5.208 min). The GABA retention times for the transformed strains (Rt = 5.279 min) and the original strain (Rt = 5.310 min) were consistent. As presented in Figure 4, 17 transformants (TG02, TG03, TG04, TG05, TG06, TG07, TG08, TG09, TG10, TG12, TG13, TG14, TG16, TG17, TG18, TG19, TG20) produced significantly higher GABA levels (17.47–134.14%) compared to the original strain. TG08, TG16, TG19, and TG18 showed increases greater than 90%. The remaining three transformants (TG01, TG11, TG15) exhibited no significant difference in GABA production compared to the original strain.

### 3.4. Transcriptomic Analysis of High MK and GABA Producing Transformed Strains

#### 3.4.1. Differential Gene Expression Analysis of Transformed Strains

**Figure 5:**
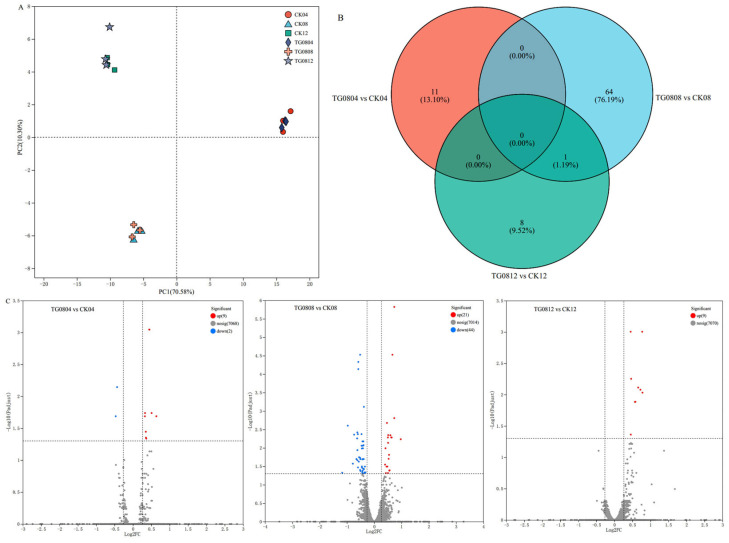
Analysis of differentially expressed genes in mycelium samples from different groups. (**A**) PCA plot of mycelium samples; (**B**) venn diagram of differentially expressed genes among groups; (**C**) volcano plot of gene expression differences in mycelium samples. Red represents upregulated genes, and blue represents downregulated genes.

RNA quality was verified (Table S1), confirming sample suitability for sequencing. RNA sequencing detected 7068 genes across the strains. PCA analysis (Figure 5A) demonstrated high repeatability and a clear separation between TG08 and CK groups, indicating that *GAD* overexpression significantly influenced gene expression.

A Venn diagram (Figure 5B) identified 65 differentially expressed genes (DEGs) in TG0808 vs. CK08, 11 in TG0804 vs. CK04, and 9 in TG0812 vs. CK12. Volcano plots (Figure 5C) illustrated upregulated and downregulated genes at different fermentation times. In TG0804 vs. CK04, 9 genes were upregulated and 2 downregulated; TG0808 vs. CK08 showed 21 upregulated and 44 downregulated genes; and TG0812 vs. CK12 exhibited 9 upregulated genes with no downregulated genes.

#### 3.4.2. Functional Annotation Analysis of Differential Genes in Transformed Strains

**Figure 6:**
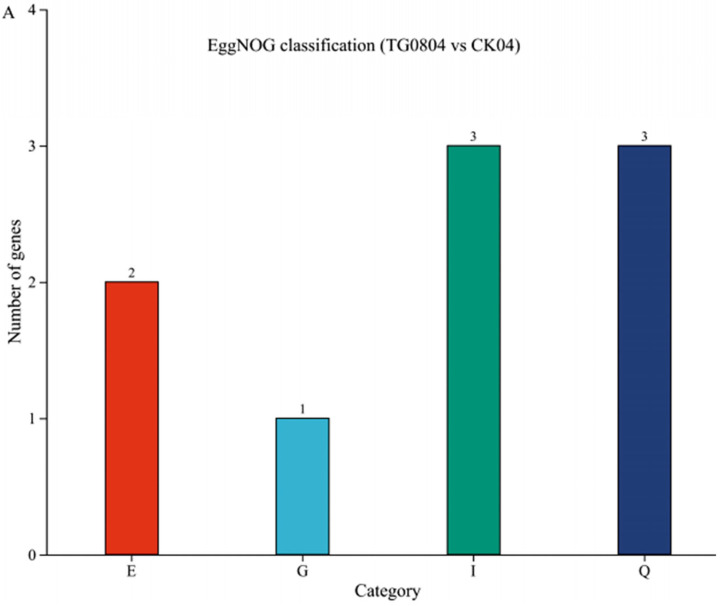

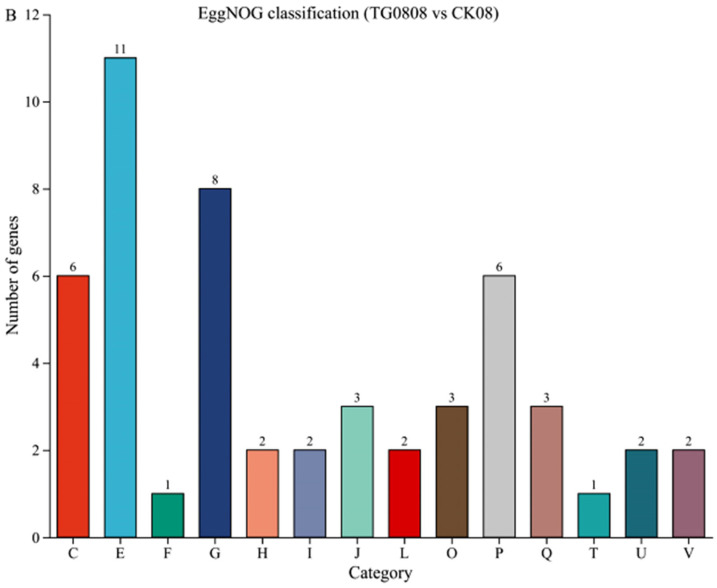

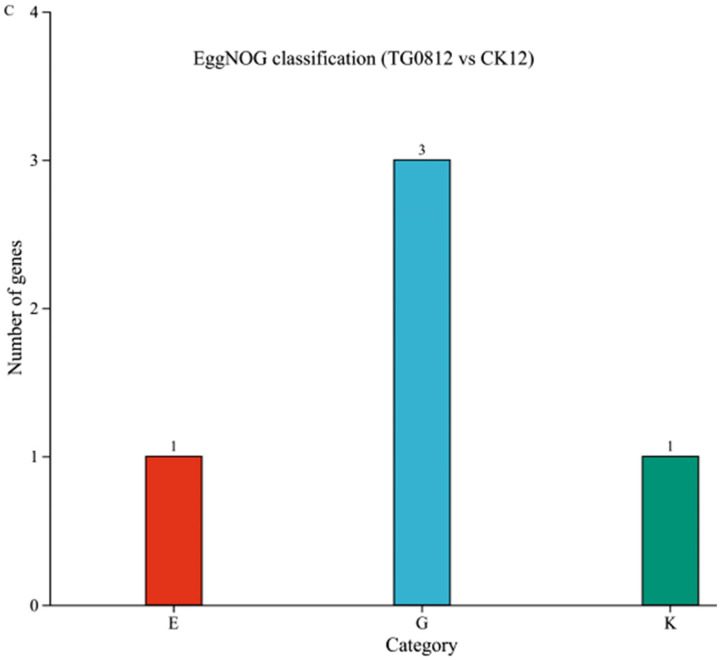

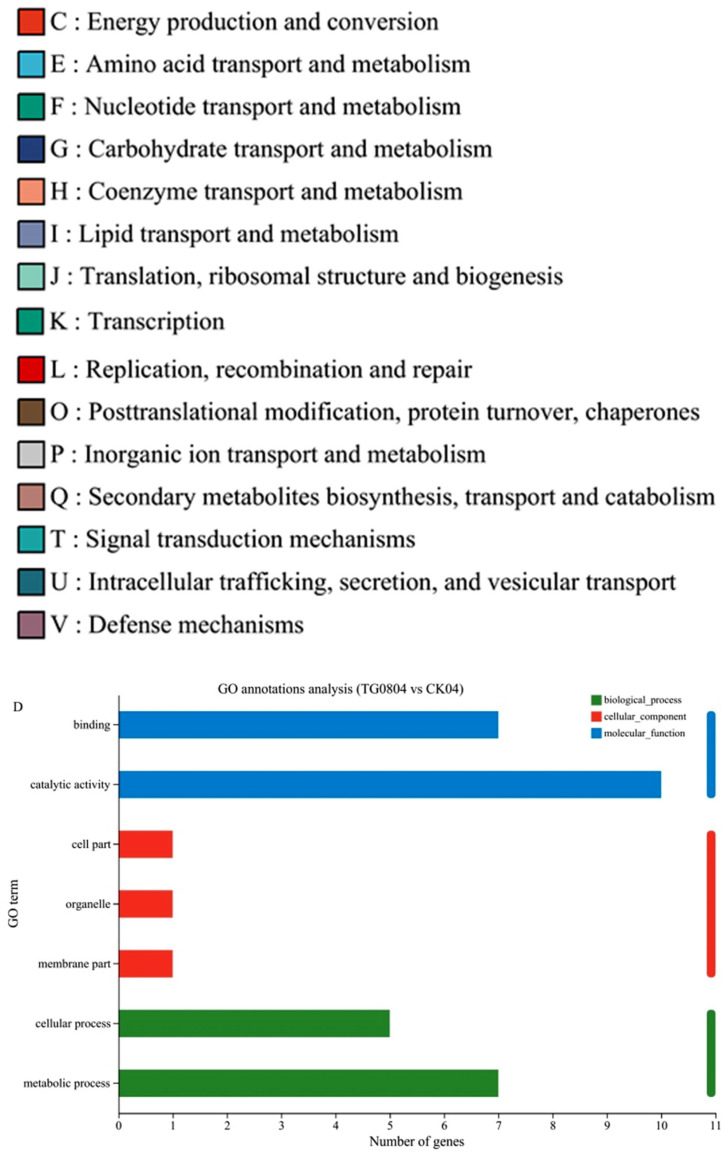

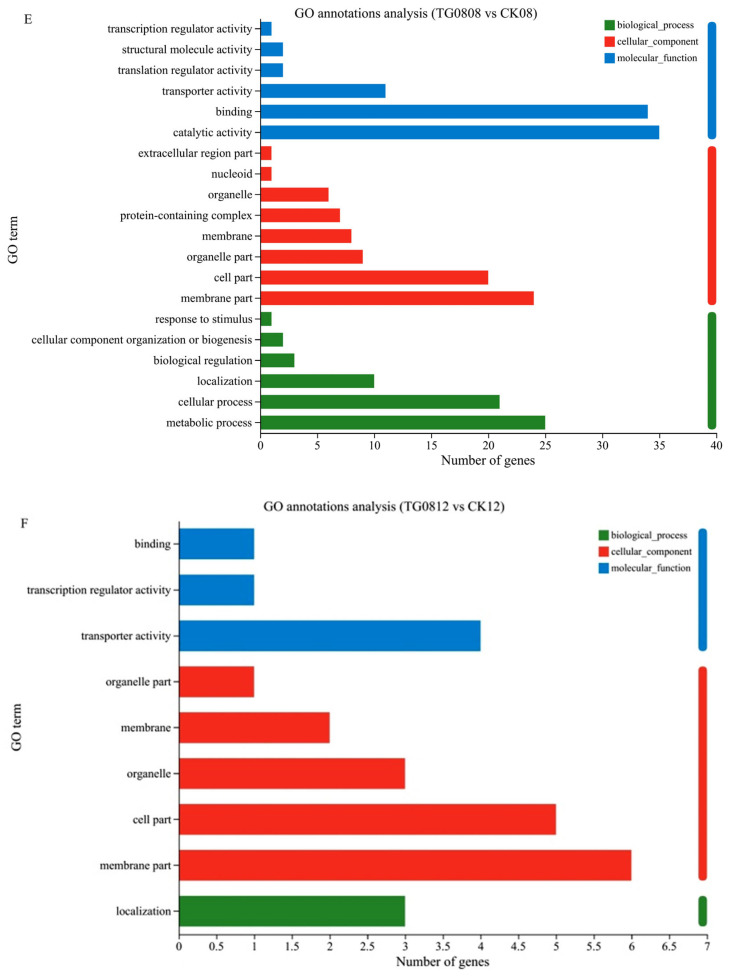

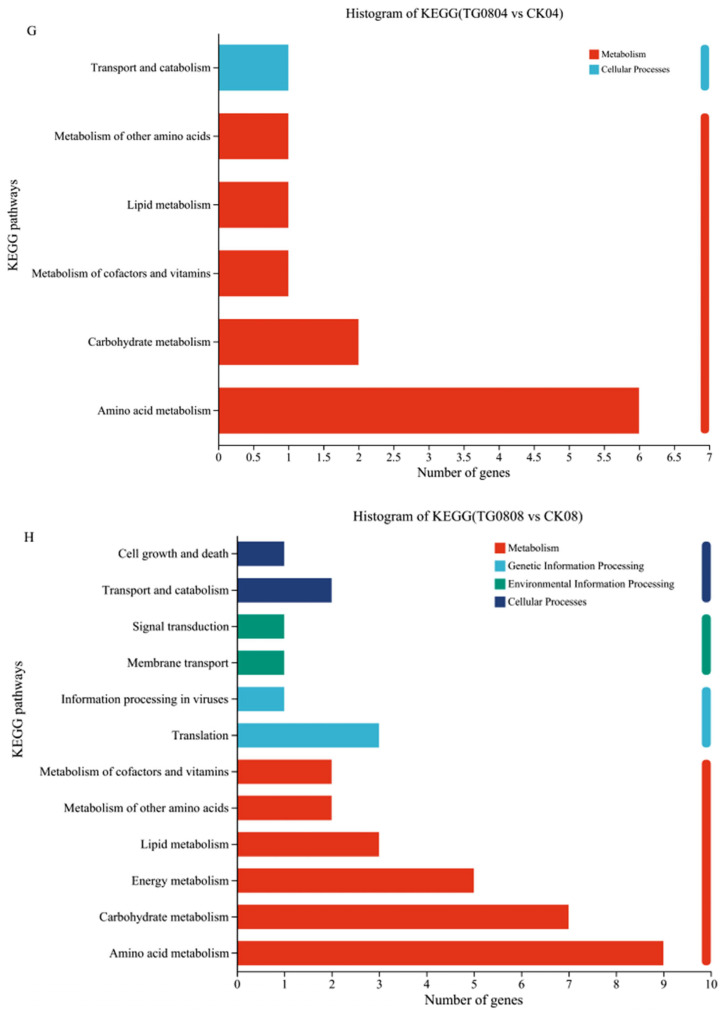

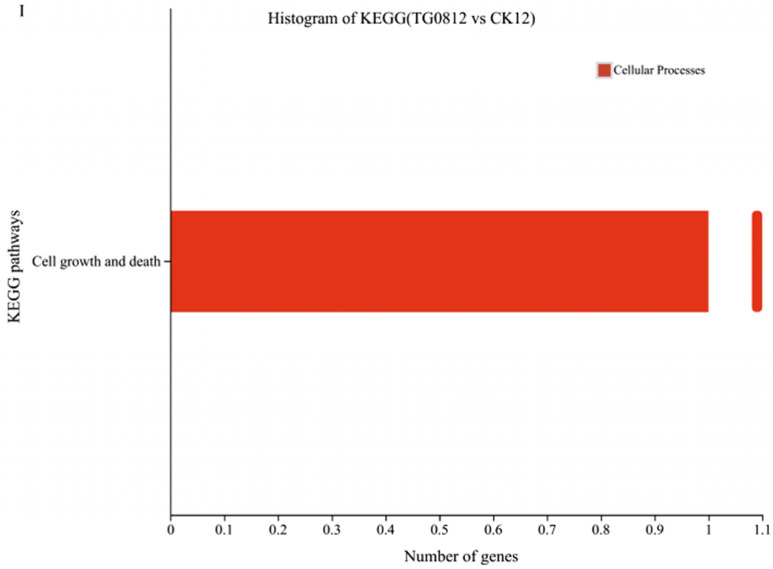
Functional annotation of differentially expressed genes in mycelium samples from each group using EggNOG, GO, and KEGG analyses. (**A**–**C**) EggNOG annotation; (**D**–**F**) GO annotation; (**G**–**I**) KEGG annotation. (**A**,**D**,**G**) TG0804 vs. CK04; (**B**,**E**,**H**) TG0808 vs. CK08; (**C**,**F**,**I**) TG0812 vs. CK12.

EggNOG functional annotation (Figure 6A–C) revealed DEGs associated with various cellular functions, including energy production and conversion, amino acid transport and metabolism, nucleotide transport and me-tabolism, carbohydrate transport and metabolism, coenzyme transport and metabo-lism, lipid transport and metabolism, translation, ribosomal structure and biogenesis, translation, replication, recombination and repair, posttranslational modification, protein turnover, chaperones, inorganic ion transport and metabolism, secondary me-tabolites biosynthesis, transport and catabolism, function unknown, signal transduc-tion mechanisms, intracellular trafficking, secretion, and vesicular transport, defense mechanisms, etc.

GO functional annotation (Figure 6D–F) categorized DEGs into biological process (BP), cellular component (CC), and molecular function (MF) categories. In BP, DEGs were linked to metabolic processes, cellular processes, response to stimulus, cellular component organization or biogenesis, biological regulation, and localization. In CC, DEGs were associated with membrane part, organelle, cellular part, membrane, extracellular region part, nucleoid, protein-containing complex, organelle part. In MF, DEGs were related to catalytic activity, binding, transporter activity, and transcriptional regulation, structural molecule activity, and translation regulator activity.

KEGG functional annotation (Figure 6G–I) showed DEGs concentrated in pathways for metabolism, cellular processes, genetic information processing, and environmental information processing. In metabolism, DEGs were related to amino acid metabolism, carbohydrate metabolism, lipid metabolism, metabolism of cofactors and vitamins, metabolism of other amino acids, and energy metabolism. In cellular processes, DEGs were associated with transport and catabolism, cell growth, and death. In genetic information processing, DEGs were involved in translation, information processing in viruses. In environmental information processing, DEGs were linked to membrane transport, and signal transduction.

#### 3.4.3. Enrichment Analysis of Differential Genes in Transformed Strains

**Figure 7:**
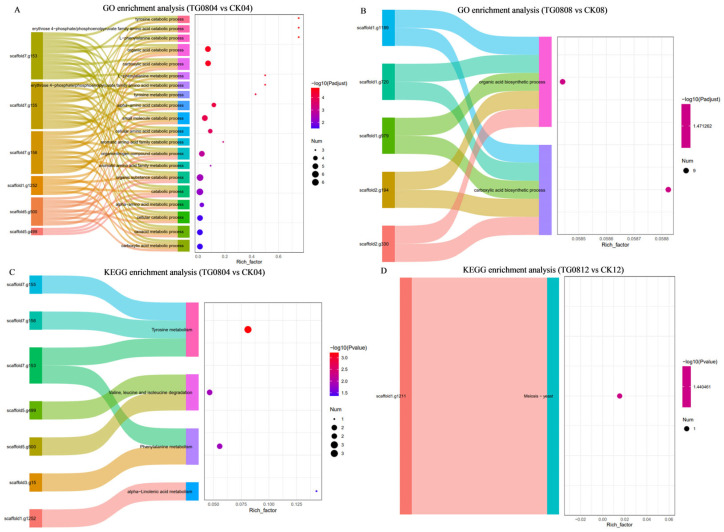
Sankey and bubble diagrams for GO and KEGG enrichment analysis of differentially expressed genes in mycelium samples from different groups. (**A**,**B**) GO enrichment; (**C**,**D**) KEGG enrichment. (**A**) TG0804 vs. CK04; (**B**) TG0808 vs. CK08; (**C**) TG0804 vs. CK04; (**D**) TG0812 vs. CK12. The bubble charts show the top 20 enriched pathways, and the Sankey diagrams highlight the top 5 differential genes sorted by *p*-value.

GO enrichment analysis (Figure 7A,B) highlighted significant pathways (*p*-value < 0.05) in TG08 vs. CK. The DEGs in TG0804 vs. CK04 were related to carboxylic acid metabolic process, oxoacid metabolic process, cellular catabolic process, α-amino acid metabolic process, catabolic process, organic substance catabolic process, aromatic amino acid family metabolic process, organonitrogen compound catabolic process, aromatic amino acid family catabolic process, cellular amino acid catabolic process, small molecule catabolic process, α-amino acid catabolic process, tyrosine metabolic process, erythrose 4-phosphate/phosphoenolpyruvate family amino acid catabolic process, L-phenylalanine metabolic process, carboxylic acid catabolic process, organic acid catabolic process, L-phenylalanine catabolic process, erythrose 4-phosphate/phosphoenolpyruvate family amino acid catabolic process, and tyrosine catabolic process (Figure 7A). The DEGs in TG0808 vs. CK08 were associated with organic acid biosynthetic process and carboxylic acid biosynthetic process (Figure 7B). No significant GO enrichment was observed for TG0812 vs. CK12 (*p*-value < 0.05).

KEGG enrichment analysis (Figure 7C,D) highlighted significant KEGG pathways (*p*-value < 0.05) in TG08 vs. CK. The DEGs in TG0804 vs. CK04 were related to tyrosine metabolism, valine, leucine and isoleucine degradation, phenylalanine metabolism, and α-linolenic acid metabolism (Figure 7C). The DEGs in TG0812 vs. CK12 were associated with meiosis–yeast pathway (Figure 7D). No significant KEGG enrichment was noted for TG0808 vs. CK08 (*p*-value < 0.05).

### 3.5. Metabolomics Analysis of High MK and GABA Production Mechanisms in Transformed Strains

#### 3.5.1. Metabolomic Detection and Multivariate Statistical Analysis of MFB Samples

LC-MS analysis identified 20,517 ion peaks, corresponding to 5560 metabolites. In positive ion mode, 10,178 ion peaks and 2921 metabolites were detected, while negative ion mode yielded 10,339 ion peaks and 2639 metabolites.

**Figure 8:**
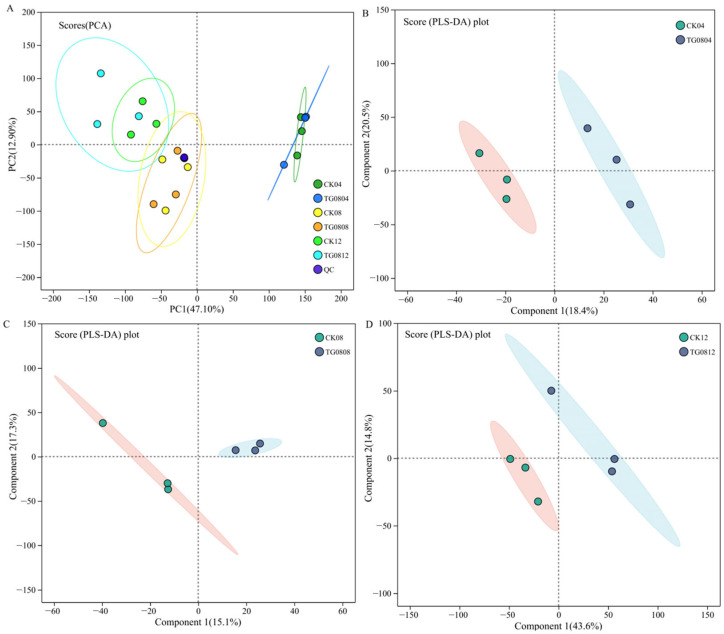
PCA and OPLS-DA analysis of metabolites in MFB samples. (**A**) PCA score plot; (**B**–**D**) OPLS-DA score plots for TG0804 vs. CK04, TG0808 vs. CK08, and TG0812 vs. CK12, respectively.

PCA and PLS-DA scores of MFB samples are shown in Figure 8. Figure 8A demonstrates small variation within groups and high biological reproducibility, confirming the reliability of the metabolomic data for further analysis. Figure 8B–D indicates clear separation between TG08 and CK groups, suggesting that *GAD* overexpression significantly altered the metabolite profiles in MFB.

The metabolomics analysis data revealed higher production of both MK and GABA in the TG08 group compared to the CK group following 12-day fermentation. Quantitative analysis revealed relative abundances of 16.80 ± 0.11 (TG08) versus 16.55 ± 0.25 (CK) for MK, and 5.33 ± 0.09 (TG08) versus 5.20 ± 0.10 (CK) for GABA. These metabolic patterns were consistent with HPLC analysis results presented in Section 3.2 and Section 3.3, validating the reliability of our untargeted metabolomics approach for subsequent differential metabolite screening and pathway enrichment analysis of *Monascus*-fermented buckwheat.

#### 3.5.2. Differential Metabolites and Pathway Enrichment Analysis of MFB Samples

**Figure 9:**
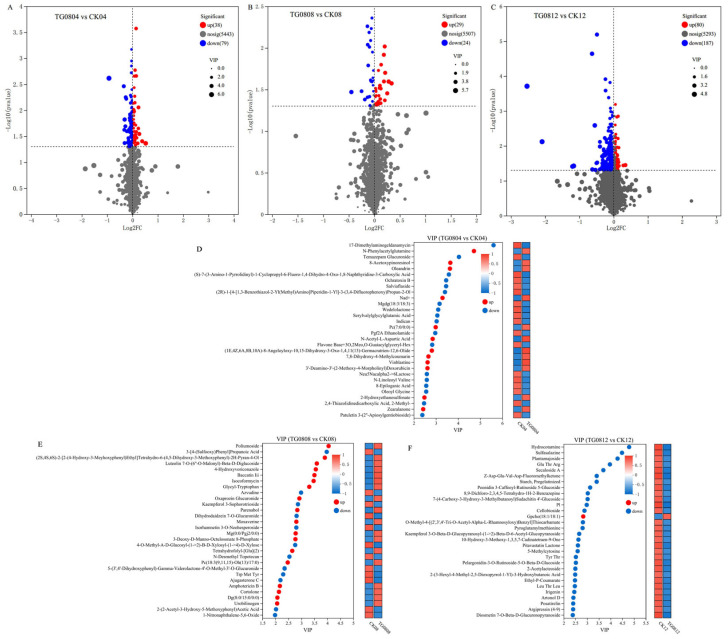
Volcano plots and VIP value analysis of differential metabolites in MFB samples. (**A**–**C**) Volcano plots; (**D**–**F**) VIP value analysis charts. (**A**,**D**) TG0804 vs. CK04; (**B**,**E**) TG0808 vs. CK08; (**C**,**F**) TG0812 vs. CK12. The VIP charts display the top 30 metabolites, sorted by VIP value. Red indicates upregulated metabolites, and blue indicates downregulated metabolites.

Volcano plots of differential metabolites between transformed strain TG08 and the starter strain CICC 5045 under identical fermentation conditions are shown in Figure 9A–C. For TG0804 vs. CK04, 117 differential metabolites were identified, with 38 upregulated and 79 downregulated. In TG0808 vs. CK08, 53 differential metabolites were observed (29 upregulated, 24 downregulated). In TG0812 vs. CK12, 267 differential metabolites were identified, including 80 upregulated and 187 downregulated metabolites.

VIP value analysis of differential metabolites is displayed in Figure 9D–F. For TG0804 vs. CK04, 12 metabolites were upregulated and 18 downregulated. In TG0808 vs. CK08, 18 metabolites were upregulated and 12 downregulated. In TG0812 vs. CK12, 1 metabolite was upregulated, while 29 were downregulated.

**Figure 10:**
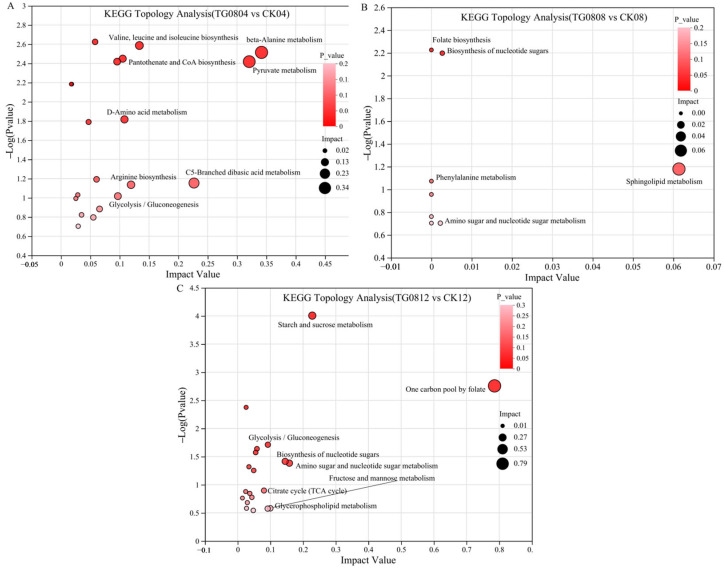
KEGG topological analysis bubble charts of differential metabolites in MFB. (**A**) TG0804 vs. CK04; (**B**) TG0808 vs. CK08; (**C**) TG0812 vs. CK12. Bubble charts highlight the top 5–8 enriched pathways based on impact value and *p*-value.

Metabolomic sets derived from differential metabolites were analyzed using KEGG pathway enrichment. For TG0804 vs. CK04, the enriched pathways included β-alanine metabolism, pyruvate metabolism, c5-branched dibasic acid metabolism, valine, leucine and isoleucine biosynthesis, arginine biosynthesis, D-amino acid metabolism, pantothenate and CoA biosynthesis, and glycolysis/gluconeogenesis (Figure 10A). For TG0808 vs. CK08, pathways such as sphingolipid metabolism, biosynthesis of nucleotide sugars, amino sugar and nucleotide sugar metabolism, folate biosynthesis, phenylalanine metabolism, and biosynthesis of cofactors were enriched (Figure 10B). For TG0812 vs. CK12, significant pathways included the one-carbon pool by folate, starch and sucrose metabolism, amino sugar and nucleotide sugar metabolism, biosynthesis of nucleotide sugars, glycerophospholipid metabolism, glycolysis/gluconeogenesis, fructose and mannose metabolism, and the tricarboxylic acid (TCA) cycle (Figure 10C).

### 3.6. Multi-Omics Joint Analysis of High MK and GABA Production Mechanisms in Transformed Strains

**Figure 11:**
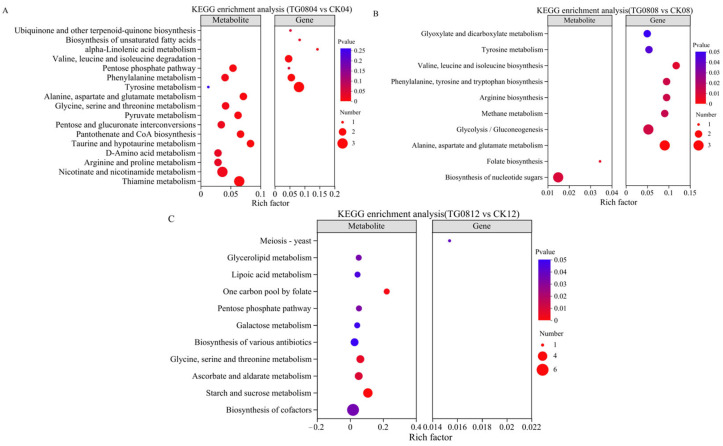
KEGG pathway enrichment map of differential genes and metabolites in MFB for each group. (**A**) TG0804 vs. CK04; (**B**) TG0808 vs. CK08; (**C**) TG0812 vs. CK12.

KEGG pathway enrichment analysis from combined transcriptomic and metabolomic data (Figure 11) revealed that in TG0804 vs. CK04, differential genes and metabolites were jointly enriched in pathways such as tyrosine metabolism, phenylalanine metabolism, pentose phosphate pathway, propanoate metabolism, and β-alanine metabolism. No common enriched pathways were identified for TG0808 vs. CK08 and TG0812 vs. CK12.

Within these five metabolic pathways, upregulated metabolites included phenylacetylglutamine, α-D-ribose-1-phosphate, and β-alanine, while pyruvate, methylglyoxal, and carnosine were downregulated. Key upregulated genes included *HPD* (4-hydroxyphenylpyruvate dioxygenase), *HGD* (homogentisate 1,2-dioxygenase), and *FAH* (fumarylacetoacetase), while *XFP* (xylulose-5-phosphate/fructose-6-phosphate phosphoketolase) was downregulated.

## 4. Discussion

In this study, we developed *GAD*-overexpressing strains, with strain TG08 achieving significant increases in both MK (47.51%) and GABA (91.11%) production compared to the parental strain CICC 5045. These findings suggest that *GAD* overexpression enhances MK and GABA synthesis, paving the way for further metabolic regulation studies.

In the tyrosine metabolism pathway, *HPD* catalyzes the conversion of 4-hydroxyphenylpyruvate to homogentisic acid, which is further degraded to fumaric acid and acetylacetoacetate through the actions of *HGD* and *FAH* [29]. In this study, upregulated genes (*HPD*, *HGD*, *FAH*) in the tyrosine metabolism pathway enhance tyrosine breakdown, producing fumaric acid and acetyl-CoA, which are crucial precursors for MK and other polyketides [30].

Additionally, fumaric acid and malic acid, key intermediates in the TCA cycle, drive energy metabolism [31]. Lee et al. [32] observed that the GABA content in germinating rice was positively correlated with organic acids in the TCA cycle, such as α-ketoglutarate, succinate, fumarate, and malate. Therefore, the upregulation of *HPD*, *HGD*, and *FAH* genes in the tyrosine metabolism pathway may not only increase acetyl-CoA for MK biosynthesis but also enhance organic acid synthesis in the TCA cycle, thus positively regulating GABA biosynthesis.

In the pentose phosphate pathway, upregulated α-D-ribose-1-phosphate may enhance downstream metabolites such as acetylsalicylate and dicarboxylates, contributing to acetyl-CoA utilization [33]. Acetyl-CoA is a crucial substrate for MK synthesis and can upregulate the expression levels of genes involved in MK synthesis, thereby enhancing MK production [34]. Li et al. [13] established an *acc* (acetyl-CoA carboxylase) gene overexpression strain to augment the supply of MK biosynthetic precursors and prominently enhancing the MK production of *Monascus*. In addition, the upregulation of α-D-ribose-1-phosphate metabolites may also indirectly affect purine metabolism, pyrimidine metabolism, and histidine metabolism pathways, thereby affecting the TCA cycle and providing more precursors for the synthesis of MK and GABA in *Monascus*.

β-alanine was upregulated in the propionic acid metabolism pathway and β- alanine metabolism pathway. Studies have shown that β-alanine is a precursor for the synthesis of pantothenic acid and CoA. Pantothenic acid is a key precursor for the biosynthesis of CoA, which is the active form of pantothenic acid in living organisms [35]. CoA is a vital cofactor for cell growth, involved in various metabolic reactions such as phospholipid synthesis, fatty acid synthesis, and the TCA cycle [36]. In this study, the upregulation of β-alanine in the *GAD* overexpressing strains can increase the level of pantothenic acid in *Monascus*, promote the synthesis of CoA, and thus enhances MK and GABA synthesis.

**Figure 12:**
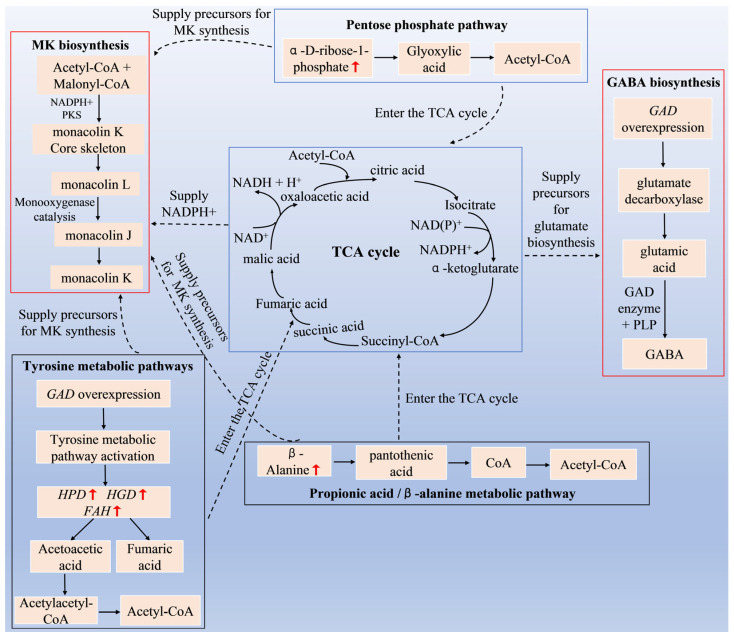
Schematic diagram of the metabolic network illustrating the multi-pathway regulatory mechanisms of MK and GABA synthesis mediated by *GAD* overexpression.

In summary, the overexpression of the *GAD* gene may promote the redirection of metabolic flux in *Monascus pilosus* and enhance its MK and GABA synthesis levels by regulating key metabolites and genes in pathways such as tyrosine metabolism, the pentose phosphate pathway, and β-alanine metabolism. This is achieved through mechanisms including enhanced TCA cycling, increased acetyl-CoA supply, and optimized energy metabolism. The proposed metabolic regulation network is illustrated in Figure 12.

## 5. Conclusions

This study successfully constructed *Monascus* strains overexpressing the *GAD* gene with high MK and GABA yields. Thirteen transformed strains exhibited 12.84–52.50% increases in MK production, while seventeen strains showed 17.47–134.14% increases in GABA production. Multi-omics analysis revealed significant enrichment in pathways such as tyrosine metabolism, phenylalanine metabolism, pentose phosphate pathway, propanoate metabolism, and β-alanine metabolism, highlighting key metabolites (e.g., α-D-ribose-1-phosphate, β-alanine), and genes (e.g., *HPD*, *HGD*, *FAH*) contributing to metabolic flux redistribution. These key metabolites and genes may become new regulatory targets and factors for future study on the biosynthesis mechanisms of MK and GABA in *Monascus*. These findings provide valuable insights for enhancing MK and GABA biosynthesis in *Monascus* strains, with potential applications in producing high-yield *Monascus*-fermented products.

## Data Availability

The original contributions presented in this study are included in the article/Supplementary Materials. Further inquiries can be directed to the corresponding authors.

## Supplementary Materials

The following supporting information can be downloaded at: https://www.mdpi.com/article/10.3390/jof11070506/s1, Figure S1. Plasmid profile of the recombinant expression vector pNeo-GAD. Figure S2. PCR and digestion identification of transformed *E. coli*. (M1) DNA Marker DL 15000; (1) Hind III/Sac I double digestion product; (2) PCR product; (M2) DNA Marker DL 2000. Figure S3. PCR identification of resistant transformants of *M. pilosus*. (M) DNA Marker DL 2000; (1–9) transformants of *M. pilosus*; (10) positive control (plasmid vector pNeo-GAD); (11) negative control (original strain CICC 5045). Table S1. RNA sample detection results.

## Abbreviations

MK, monacolin K; GABA, γ-aminobutyric acid; MPs, *Monascus* pigments; GAD, Glutamate decarboxylase; HPD, 4-hydroxyphenylpyruvate dioxygenase; HGD, homogentisate 1,2-dioxygenase; FAH, fumarylacetoacetase; PHAA, phenylacetate 2-hydroxylase; ACX, acyl-CoA oxidase; XFP, xylulose-5-phosphate/fructose-6-phosphate phosphoketolase; RYR, red yeast rice; MFB, *Monascus* fermented buckwheat; HPLC, high-performance liquid chromatography; DEGs, differentially expressed genes; BP, biological process; CC, cellular component; MF, molecular function.
