# Supplementary material for: Enhancing Monacolin K and GABA Biosynthesis in Monascus pilosus via GAD Overexpression: Multi-Omics Elucidation of Regulatory Mechanisms
Source: J Fungi (Basel). 2025 Jul 4;11(7):506. doi: 10.3390/jof11070506 (PMC12295812; doi:10.3390/jof11070506)
Supplement: Supplementary file 1 [file jof-11-00506-s001.zip › jof-3661199-supplementary.pdf]

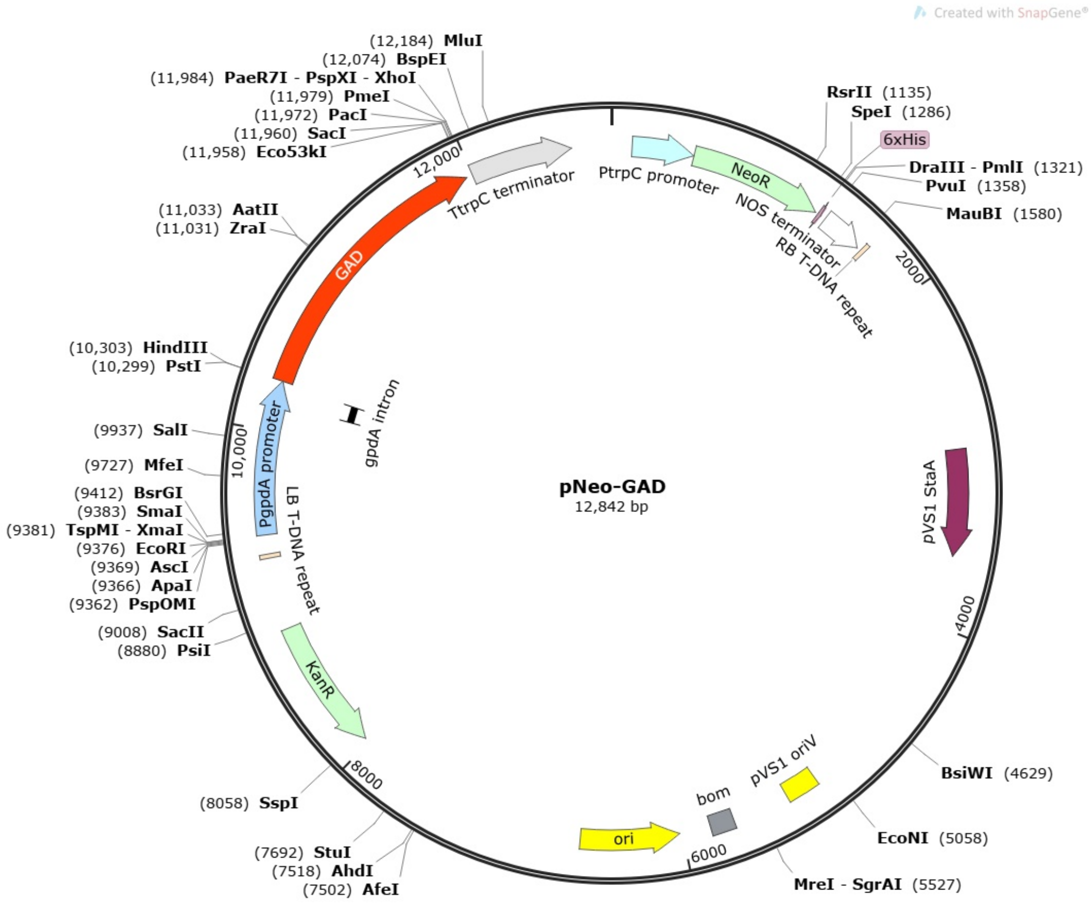

Figure S1. Plasmid profile of the recombinant expression vector pNeo-GAD.

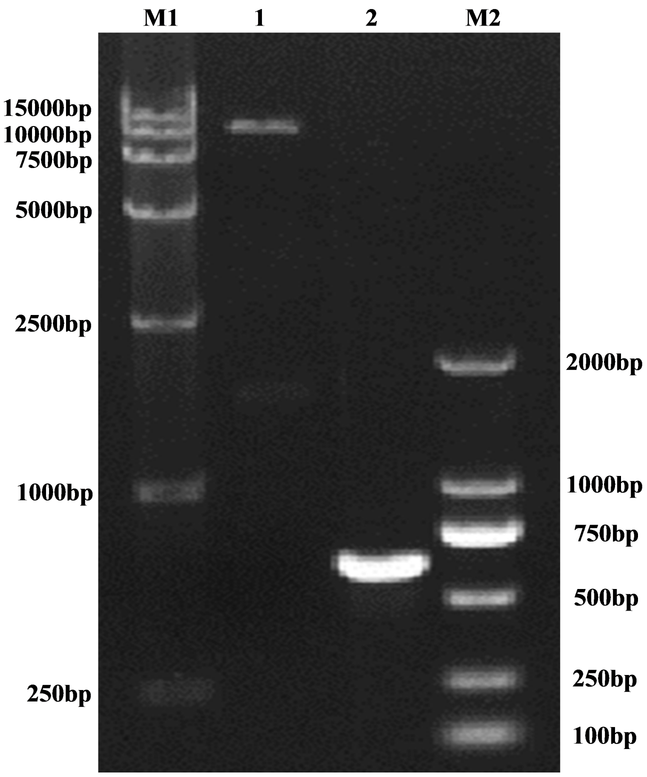

Figure S2. PCR and digestion identification of transformed *E. coli*. (M1) DNA Marker DL 15000; (1) Hind III/Sac I double digestion product; (2) PCR product; (M2) DNA Marker DL 2000.

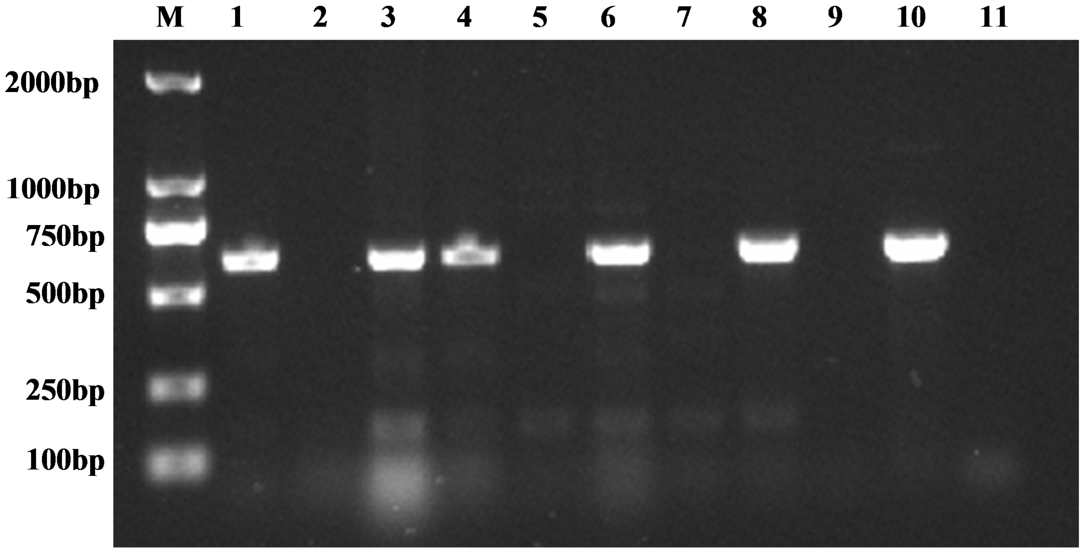

Figure S3. PCR identification of resistant transformants of *M. pilosus*. (M) DNA Marker DL 2000; (1–9) transformants of *M. pilosus*; (10) positive control (plasmid vector pNeo-GAD); (11) negative control (original strain CICC 5045).

Table S1. RNA sample detection results.

RNA samples	Concentration (ng/μL)	Total amount (μg)	RQN	Quality testing results
CK0401	137.03	4.796	10	A
CK0402	234.36	8.203	10	A
CK0403	134.14	4.695	10	A
TG080401	152.07	5.322	10	A
TG080402	103.27	3.615	10	A
TG080403	142.6	4.991	10	A
CK0801	117.66	4.118	10	A
CK0802	165.62	5.797	10	A
CK0803	131.6	4.606	10	A
TG080801	153.98	5.389	10	A
TG080802	120.84	4.229	10	A
TG080803	154.64	5.412	10	A
CK1201	135.5	4.742	10	A
CK1202	160.95	5.633	10	A
CK1203	169.17	5.921	10	A
TG081201	144.61	5.061	10	A
TG081202	194.59	6.811	10	A
TG081203	143.3	5.015	10	A

RQN: RNA Quality Number.
